# Co-existence of Alport syndrome and C3 glomerulonephritis in a proband with family history

**DOI:** 10.1186/s40001-021-00543-5

**Published:** 2021-07-08

**Authors:** Yin Ding, Xuanli Tang, Yuanyuan Du, Hongyu Chen, Dongrong Yu, Bin Zhu, Bohan Yuan

**Affiliations:** Department of Nephrology (Key Laboratory of Management of Kidney Disease in Zhejiang Province), Hangzhou TCM Hospital Affiliated to Zhejiang Chinese Medical University, Tiyuchang Road 453, Hangzhou, 310007 People’s Republic of China

**Keywords:** Rare renal disease, Alport syndrome, C3GN, *CFHR5* p.Val170Met

## Abstract

**Background:**

Alport syndrome and C3 glomerulonephritis (C3GN) are rare kidney diseases, frequently responsible for familial haematuria, proteinuria, and renal impairment. With the rapid development of molecular genetic testing, Alport syndrome causes have been restricted mostly to variants in the *COL4A5* or *COL4A3*/*COL4A4* genes. Moreover, a broad range of genetic contributors in the complement and complement-regulating proteins are definitely implicated in the pathogenesis of C3GN.

**Methods:**

We sought a family with persistent microscopic haematuria associated with renal failure. Clinicopathologic and follow-up data were obtained, and molecular genetic testing was used to screen for pathogenic variants.

**Results:**

We describe a three-generation family with Alport syndrome showing a dominant maternal inheritance. Notably, renal biopsy showed the concurrent histological evidence of C3GN in the proband harbouring an uncommon heterozygous variation in *CFHR5*, c.508G > A. The alteration leads to replacement of a highly conserved residue at position 170 of the β-strand subunit of *CFHR5* (p.Val170Met). In silico analysis showed that the variation was predicted to deregulate complement activation by altering the structural properties and enhancing C3b binding capacity to compete with Complement Factor H (CFH), which was in line with experimental data previously published.

**Conclusions:**

The comorbidity findings between Alport syndrome and C3GN indicate an underlying overlap and require further study.

**Supplementary Information:**

The online version contains supplementary material available at 10.1186/s40001-021-00543-5.

## Background

In China, a rare disease is defined as a condition that affects fewer than 500,000 people [1]. Approximately 80% of rare diseases have an identified genetic background with substantial geographic or ethnic variations in incidence [2]. 

Genetic analysis was first used in nephrology for the identification of a causal mutation for Alport syndrome in 1990 [3]. Alport syndrome is a rare hereditary disorder in which the glomerular basement membrane (GBM) has an abnormal collagen IV composition. It is characterized by haematuria, progressive renal failure, sensorineural deafness, anterior lenticonus and retinal flecks [4]. To date, the causes of familial haematuria nephropathies have been restricted mostly to variants in the *COL4A5* or *COL4A3/COL4A4* genes, responsible for X-linked or autosomal inheritance [5–7]. Moreover, analysis of data suggests that familial microscopic haematuria attributable to the complement factor H-related protein 5 (*CFHR5*) gene is also associated with a rare renal disease termed C3 glomerulonephritis (C3GN) [8–12], defined as glomerular C3 position in the absence of significant amounts of immunoglobulin, C1q, and C4, and without intramembranous glomerular basement membrane dense deposits.

Herein, we report a family with dominant maternal transmitted inheritance, in which five out of eight members (I:1, II:1, II:4, III:1, and III:2) exhibited microscopic haematuria and proteinuria and two out of the 5 patients (II:1 and III:1) developed end-stage renal disease (ESRD). The diagnosis of Alport syndrome was confirmed in the proband (III:1), his mother (II:1), and his maternal aunt’s daughter (III:2) based on all available data. Furthermore, renal biopsy showed the comorbidity of Alport syndrome and C3GN in the proband carrying an uncommon heterozygous c.508G > A (p.Val170Met) variant in the *CFHR5* gene. Furthermore, the functional effect of the p.Val170Met variation was exhaustively evaluated. 

## Methods

### Family pedigree and clinical investigations

In this study, a family with persistent microscopic haematuria associated with renal failure was recruited from Hangzhou, Zhejiang Province, China. All affected members were regularly followed up at the out-clinic until May 12, 2020. A brief history and physical examination were performed and blood, urine, skin, kidney samples were collected for determination of serum creatinine concentration, estimated glomerular filtration rate (eGFR), urinalysis, C3/C4 levels and histological manifestation.

### Renal and skin biopsies processing

Biopsy specimens for light microscopy were fixed in alcohol formaldehyde acetic solution, embedded in paraffin, and cut into 2-μm-thick sections. Sections were stained with Masson trichrome, hematoxylin and eosin, periodic acid–Schiff, and silver methenamine. Samples for immunofluorescence study were stained with fluorescein isothiocyanate-conjugated polyclonal antibodies to human IgA, IgG, IgM; C3; light chains (*κ* and *λ*); C4, C1q; and α5 chains of type IV collagen. Antigen was retrieved by EDTA solution as well as gastric enzyme, and Elivision method was applied in IHC detection. Specimens for electron microscopy were fixed in glutaraldehyde and embedded in epon. All reports were reviewed by two pathologists.

### Targeted exon sequencing and Sanger sequencing

Genomic DNA was isolated from peripheral blood of a three-generation pedigree of eight members using the Wizard Genomic DNA Purification Kit (Promega, USA) following the manufacturer’s instructions. Coding exons reference sequences of the individuals (II:1, II:2, III:1 and III:2) were targeted for the Illumina high-throughput sequencing, and reads were aligned to the human genome reference sequence (hg19/GRCh37). The approximately 95% of reads were mapped to the target regions at an average of 20 × . We examined copy number, rare, and common variants. All the disease-related sites were selected and Sanger sequencing was further performed in all eight members. Primers were indigenously designed using the primer premier 5.0 program (Lalitha, 2000) and shown in Additional file 1: Table S1. The purified PCR products were directly sequenced using an ABI BigDye Terminator v3.1 Cycle Sequencing Kit. The analyses were completed on an ABI-3500Dx Genetic Analyzer (Applied Biosystems).

### In silico evaluation of pathogenicity

The ProtParam tool was used to compute various physical and chemical parameters including the molecular weight and theoretical pI. The complete amino acid sequence data of the human protein *CFHR5* (GenBank Accession: AAI11774.1) were obtained from the NCBI (National Center for Biotechnology Information) and alignments done by the EMBL-EBI (European Bioinformatics Institute). The SIFT (Sorting Intolerant From Tolerant), SNAP (Screening for Non-acceptable Polymorphisms), PolyPhen-2 (Polymorphism Phenotyping v2), and Mutation Taster were utilized to evaluate possible biologic effects of genetic aberration impact on protein structure.

### Variant interpretation

In 2015, standards and guidelines for the interpretation of sequence variants were updated by the American College of Medical Genetics and Genomics (ACMG), the Association for Molecular Pathology (AMP) and the College of American Pathologists (CAP) [13]. This report recommends the use of specific standard terminology: ‘pathogenic’, ‘likely pathogenic’, ‘uncertain significance’, ‘likely benign’, and ‘benign’ to describe variants.

In brief, an allele frequency in a control population that is greater than expected for disorder or with lack of segregation among affected individuals was considered strong support for a benign interpretation or, if over 5%, it is considered as ‘benign’. Novel or rare variants that lead to splicing defect or amino acid change coupled with multiple lines of computational evidence, or cosegregate with disease, were classified as ‘pathogenic’ or ‘likely pathogenic’, and variants with well-established in vitro or in vivo functional studies that indicate a damaging effect were labelled ‘pathogenic’. Variants of ‘uncertain significance’ are more common: other criteria shown above are not met or the criteria for benign and pathogenic are contradictory. 

### Homology modelling of human *CFHR5*/C3b complex

Docking procedure has been described in more detail previously [14]. Starting from residues 23–569, the 3D homology models of *CFHR5* were first generated by iterative threading assembly refinement (I-TASSER) server [15], where the one with the highest C-score was selected to be further refined by Fragment-Guided MD simulation (FG-MD) [16]. Potential energy of refined protein was calculated by “Calculate Energy” protocol of Discovery Studio (DS) 3.0. The crystal structure of ligand C3b (PDB ID: 2WII) was taken from protein data bank (PDB).

By applying “Prepare Protein” protocol of DS, energy minimization was performed to clean the protein molecule by adding missing atoms, inserting missing loops, assigning charges and fixing CHARMm force fields. A total of 2000 docking poses were generated by ZDOCK (CHARMm-based DOCKER) protocol and incorporated. The top1 model was collected as the probable complex structure. Hydrogen-bonding networks can be displayed by Molecular Operating Environment (MOE). Binding free energy of protein–protein interaction was estimated by the Calculate Mutation Energy (Binding) protocol within DS.

## Results

### Clinical presentation and follow-up

**Fig. 1:**
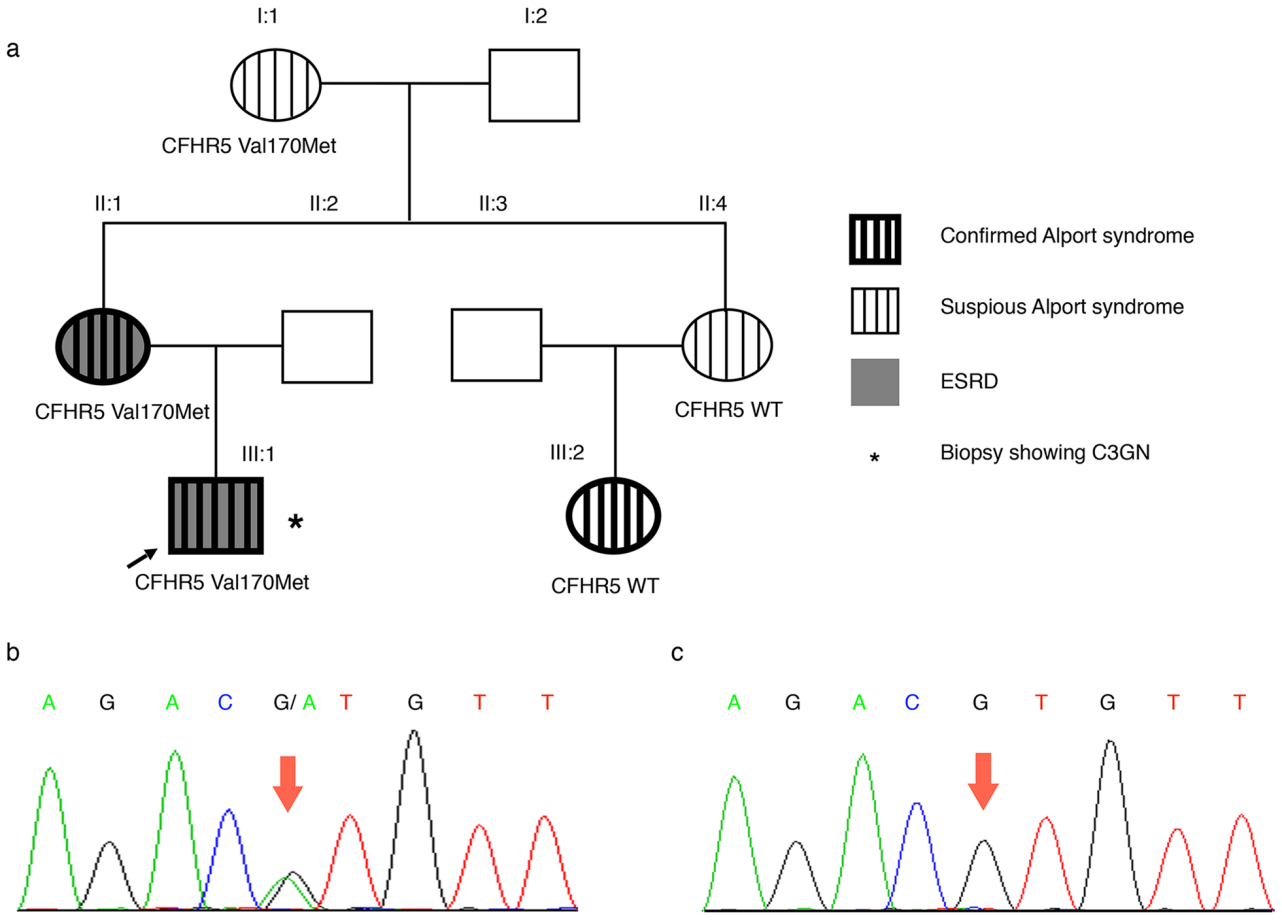
Family pedigree and Sanger sequencing chromatograms. **a** Pedigree structure of the family with Alport syndrome. Squares and circles denote males and females, respectively. Roman numbers indicate generations. Arrow indicates the proband (III:1). Family members carrying the heterozygous *CFHR5* genetic variant (c.508G > A; Val170Met) are highlighted. Individuals II:4 and III:2 do not carry the Val170Met variant. **b** Sequence electropherogram shows a heterozygous *CFHR5* c.508G > A (p.Val170Met) variation (arrow) in individual I:1, II:1, and III:1. **c** Sequence electropherogram shows the negative control

We investigated a three-generation Han Chinese family characterized by persistent microscopic haematuria associated with renal failure (Fig. 1a). The proband (III:1), a 11-year-old boy, was admitted to the hospital for haematuria and proteinuria. On admission, 24-h urine protein was 6.36 g/day and serum albumin was 24.4 g/L. Microscopic urinary analysis showed urine sediment containing +  +   to   +  +  + red cells per high-power field. His blood urea nitrogen level was 15.73 mmol/L, serum creatinine level was 266 μmol/L and eGFR by CKD-EPI equation was 30.3 mL/min. Bilateral renal ultrasound revealed abnormal changes with strong and unevenly distributed echoes, and unclear demarcation between the renal cortex and parenchyma. Sensorineural hearing impairment and vision abnormalities, as well as other clinical signs, were not detected.

During follow-up, the proband was started on haemodialysis 3 months later and received a kidney transplant after one year. The proband (III:1) had a family history of haematuria, as his mother (II:1) presented with haematuria, proteinuria, and developed ESRD before the age of 33 years. His maternal grandmother (I:1), maternal aunt (II:4), and aunt’s daughter (III:2) also had urinary abnormalities characterized by asymptomatic haematuria and proteinuria with normal eGFR. His father (II:2), his maternal grandfather, and aunt’s husband were phenotypically normal.

**Fig. 2:**
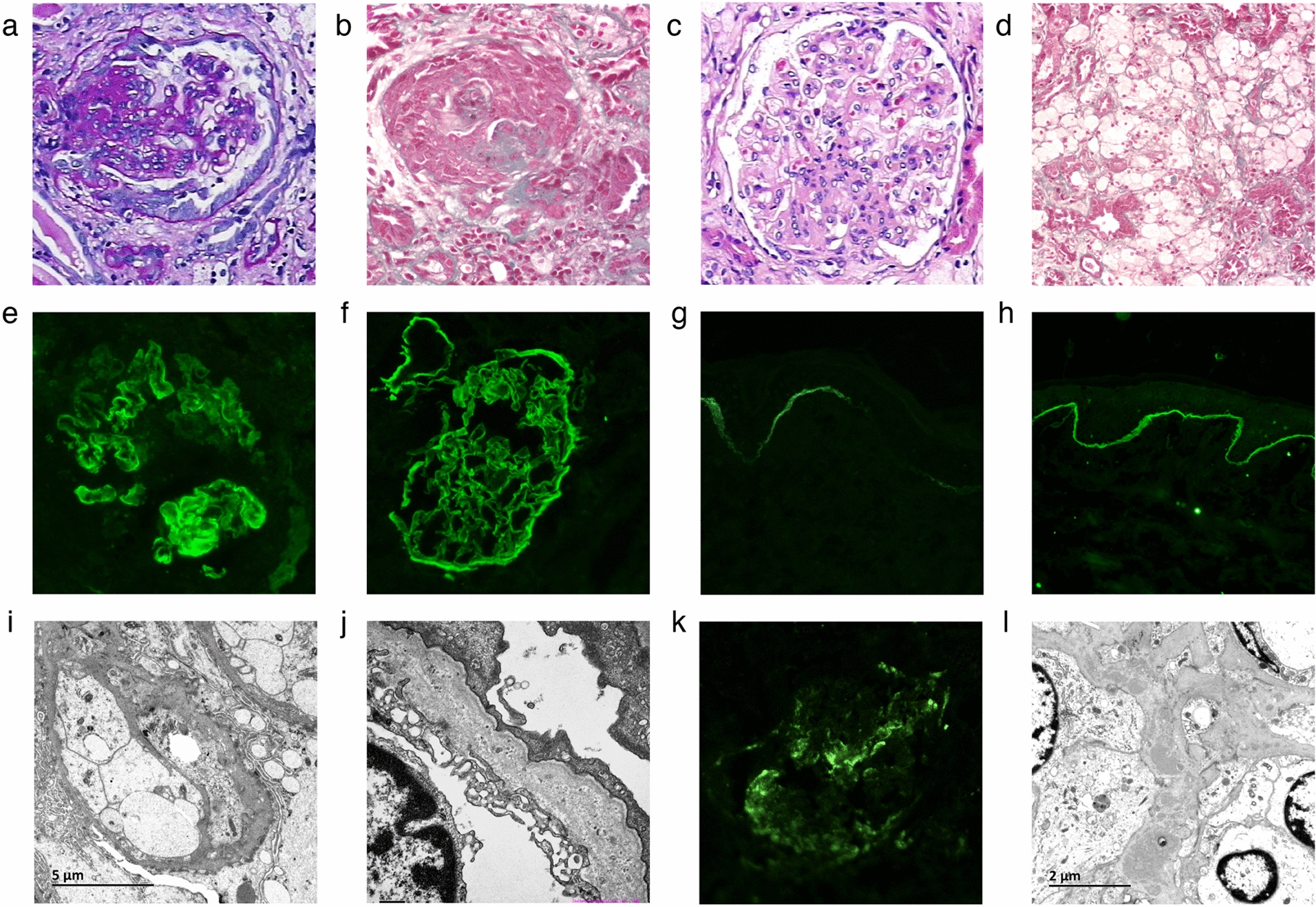
The pathological features of kidney and skin samples in the proband (III:1), his mother (II:1), and His affected aunt’s daughter (III:2). Light micrograph: the proband shows segmental sclerosis (**a**, PAS × 200), crescent formation (**b**, MA × 200), diffuse and global mild-moderate mesangial proliferation (**c**, HE × 200), and interstitial fibrosis/tubular atrophy containing lipid-laden foam cells (**d**, MA × 200). Immunofluorescent stain: the proband reveals partial loss for the collagen IV a5 chain in GBM and complete absence in Bowman’s capsule and distal tubule basement membrane (**e**, IF × 400), compared with control (**f**, IF × 400). A skin biopsy of his mother demonstrates segmental absence of the collagen IV a5 chain within the epidermal basement membrane (**g**, IF × 200), compared with control (**h**, IF × 200). Electron micrograph: segmental uneven thickness with unsmooth spiculation of the dense layer in the GBM is found in the proband (**i**,  × 6000), and uneven thickness with a laminated appearance is shown in his aunt’s daughter (**j**, × 15,000). Remarkably, a marked C3 staining is shown in the proband to locate along the mesangium by immunofluorescence analysis (**k**, IF × 200) with the corresponding electron-dense deposits under the electron microscopy (**l**, × 10,000)

To confirm the clinical diagnosis, renal biopsy of the proband was performed. The biopsy sample contained 15 glomeruli on light microscopy: global glomerular sclerosis in four glomeruli, segmental sclerosis in three other glomeruli (Fig. 2a), and four crescents were found (Fig. 2b). Diffuse and global mild–moderate proliferation of mesangial cells and mesangial matrix were shown in the remaining glomeruli (Fig. 2c). Besides, interstitial fibrosis/tubular atrophy containing lipid-laden foam cells were obvious (Fig. 2d). Immunofluorescence studies showed patchy GBM expression of a5 (IV) collagen and complete absence in Bowman’s capsule and distal tubular basement membrane (BM) (Fig. 2e), compared with control (Fig. 2f). A skin biopsy was performed on his mother (II:1), which revealed that there was segmental absence of the collagen IV a5 chain within the epidermal basement membrane (Fig. 2g), compared with control (Fig. 2h).

Electron microscopy identified pathological characteristics accompanied with noticeably segmental uneven thickness with unsmooth spiculation and suspected lamination of the dense layer within the basement membrane in the proband (Fig. 2i). His affected aunt’s daughter (III:2) had a renal biopsy at age 13, showing segmental uneven thickness with a laminated appearance of the dense layer in GBM (Fig. 2j).

Notably, a marked C3 deposition (++ to +++) in the mesangial region was detected in the proband under immunofluorescence microscopy (Fig. 2k), with the corresponding lump electron-dense deposits and mild–moderate mesangial proliferation observed with electron microscopy (Fig. 2l). Immunofluorescence examination revealed none or trace positivity of IgA, IgG, IgM, C4, C1q, Fib, *κ* and *λ* fragments.

### Findings on targeted exome-based next-generation sequencing (NGS) and Sanger sequencing

**Fig. 3:**
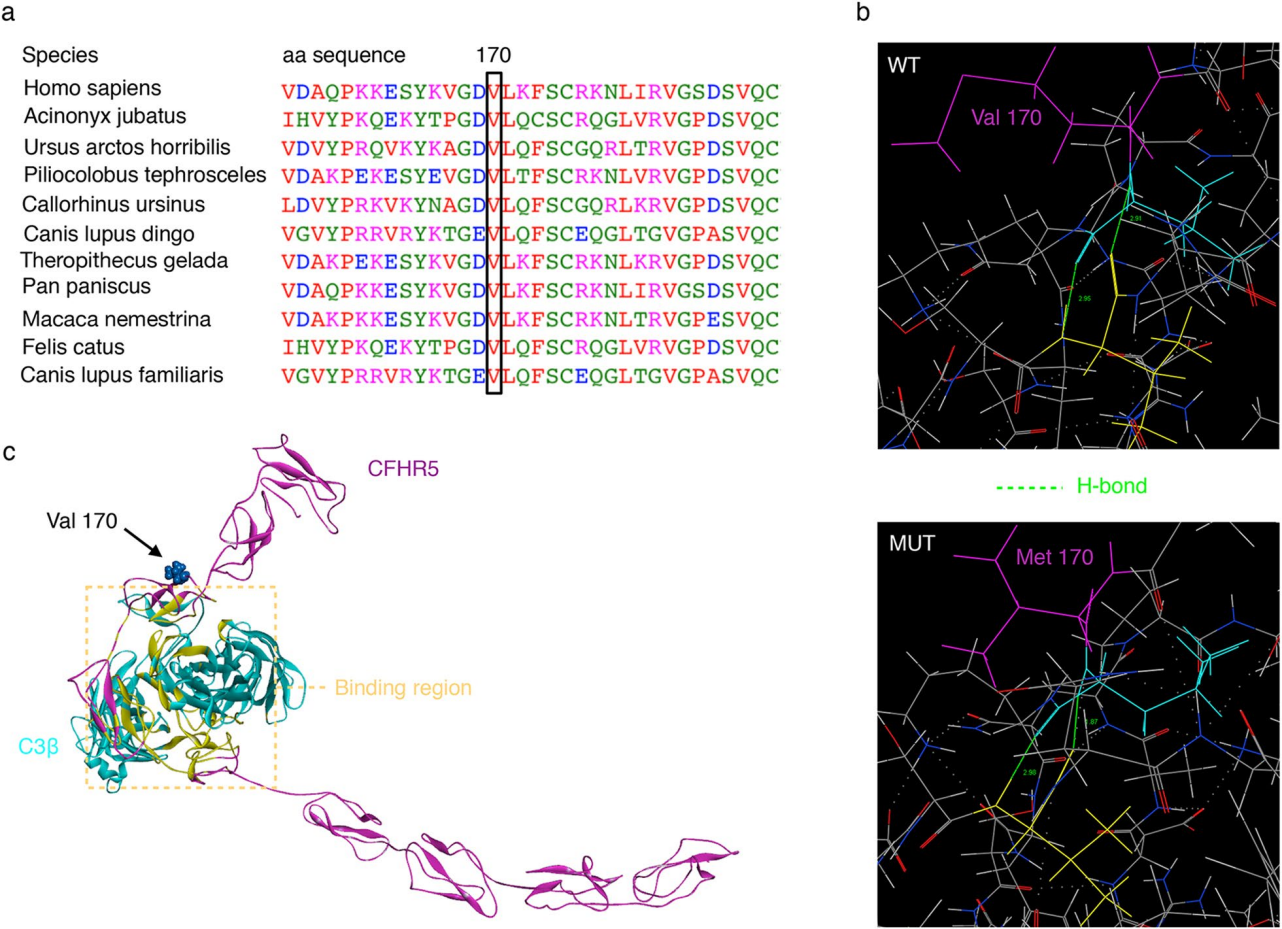
Functional characterization of the *CFHR5* p.Val170Met variant. **a** Alignment of the *CFHR5* protein in different species shows the conservation of the V170 residue. The concerned amino acids are boxed. **b** Generated models show the discrimination of local intramolecular hydrogen-bonding interactions between Val170 and Met170 by MOE. Hydrogen bonds are shown with a fluorescent green dotted line representation. **c** Structure of *CFHR5* in complex with the ligand C3b-β chain (C3β) were generated with the DS 3.0. *CFHR5* is denoted by pink, C3β (residues 1–642, PDB 2WII) is shown in turquoise. The binding regions of the *CFHR5*/C3β complex are presented in yellow. The Val residue 170 of *CFHR5* is highlighted with a CPK model

To make a precise diagnosis for the true pathogenic mechanism affecting the family, we performed whole exome-based NGS and further confirmed the results with Sanger sequencing. Genetic analysis identified the same heterozygous c.508G > A coding variant (p.Val170Met) in exon 6 of the *CFHR5* gene in three affected individuals: the proband (III:1), his mother (II:1) and his maternal grandma (I:1) (Fig. 1b). The variant presents low frequency in the general population (1000 Genomes: minor allele frequency [MAF] = 0.1%), and the mutated A allele carriers are all heterozygous. Compared across ethnicities, this variant is more frequent in East Asians (1000 Genomes: 0.6%), especially in the Chinese Han population (1000 Genomes: 1%). The nonsynonymous alteration leads to the replacement of a valine, strictly conserved among organisms (Fig. 3a), by a methionine residue. The screening with four publicly available programs (SIFT, SNAP, PolyPhen-2, and Mutation Taster) independently predicted that the replaced amino acid was “damaging” to the protein structure/function (SIFT score 0.004; SNAP score 20; PolyPhen2 score 0.999; Mutation Taster: might be affected).

No suspicious disease-causing variants were detected in the genes *COL4A3*, *COL4A4*, or *COL4A5* that encode the α3, α4, or α5 chains of type IV collagen, respectively.

### In silico functional prediction for *CFHR5* c.508G > A, p.Val170Met

*CFHR5* is a single-chain polypeptide composed of nine complement control protein domains (also known as CCPs). The residue p.Val170Met in *CFHR5* occurs in the β-strand region of the CCP3. The isoelectric point (pI) is found to be the same for both wild type and mutant proteins (pI 6.8). Molecular weight of the mutant (64.45 kDa) protein is similar to that of wild-type protein (64.42 kDa). In the native structure, the Val residue, located in the buried surface, is not involved in any intramolecular interactions. As substituted by Met170, the original intramolecular hydrogen-bonding distance between the side chain of its neighbouring residues (Leu171 and Val187) changes (Fig. 3b). DS 3.0 analysis predicted the potential energy of the mutant-type protein is − 15180.97 kcal/mol compared to − 15133.86 kcal/mol for the wild-type one, which implied that p.Val170Met could lead to an increase in conformational stability of the *CFHR5* protein.

As with Complement Factor H (CFH) and other Complement Factor H Related Proteins (CFHRs), *CFHR5* regulates the complement cascade by binding and interacting with the macromolecular protein ligand C3b. The C3b fragment is a glycoprotein composed of the modified C3-α chain (C3α') and the intact C3-β chain (C3β). We then simulated the probable native *CFHR5*/C3β complex structure. As it can be seen in Fig. 3c, the three-dimensional (3D) model demonstrated that Val170 is not the binding site for protein C3b. However, further binding free-energy calculation showed that p.V170M slightly increases the binding affinity of *CFHR5*/C3β complex by − 0.76 kcal/mol.

## Discussion

Alport syndrome is characterized by haematuria, renal failure, and extra-renal alterations, such as: hearing loss, lenticonus, and retinal flecks [4–7]. The disease is caused by changes in the collagen type IV chains, resulting in damage to the basement membrane of several organs. Approximately 85% of families have X-linked inheritance with mutations in *COL4A5* gene [3, 17–19], and most of the other affected families have autosomal recessive disease with alterations in both copies of COL4A3 or COL4A4 [20–22]. Autosomal dominant inheritance is very rare and results from heterozygous COL4A3 or COL4A4 variants.

In our study, we comprehensively examined the history of a three-generation family. The proband (III:1) presented progressive deterioration of renal function with persistent haematuria and proteinuria, and underwent a kidney transplant at age 12. His family history was significant, as her mother progressed to ESRD, over a 10-year follow-up period of haematuria and proteinuria. Besides, his maternal grandma (I:1), maternal aunt (II:4), and aunt’s daughter (III:2) had asymptomatic microscopic haematuria (< 30/HP) and proteinuria (< 0.5 g/d) with normal renal function. Characteristic pathological changes were observed in renal biopsy specimens from the proband (III:1) and his maternal aunt’s daughter (III:2), including segmental uneven thickness with lamellation and splitting of the dense layer in GBM under electron microscopy. In addition, immunostaining showed nearly complete loss for a5 (IV) collagen chain in GBM and complete absence in Bowman’s capsule and distal tubule basement membrane in the proband. His mother (II:1) exhibited segmental distribution of a5 (IV) expression in the skin BM. According to the diagnostic algorithm published in 2013 [7], the diagnosis of Alport syndrome was established in the proband (III:1), and his mother (II:1) and his maternal aunt’s daughter (III:2) based on pedigree study, clinical manifestation, and skin/renal biopsy.

The *COL4A3* and *COL4A4* genes are located on chromosome 2, while the *COL4A5* gene is located on the X chromosome, these genes encode the collagen IV a3, a4, and a5 chains, respectively. In normal individuals, the collagen IV a3a4a5 chains are highly expressed and co-distributed within the mature kidney (GBM and distal tubular BM), cochlea, and eye, and the collagen IV a5a5a6 network occurs in the skin BM and kidney (Bowman’s capsule) [5]. X-linked Alport syndrome accounts for the majority of Alport syndrome cases, arising from mutations in the *COL4A5* gene. In the proband and his mother, morphologic phenotype demonstrated reduced and depleted expression of the collagen IV a5 chain in kidney and/or skin. Further clinical and genealogical study indicated that the affected proband (III:1) had much more severe disease than the females [his mother (II:1), maternal grandma (I:1), maternal aunt (II:4) and aunt’s daughter (III:2)] in the family, and male-to-male transmission was absent. Hence, the mode of inheritance was suspected to be X-linked.

Molecular genetic testing is one of the criteria for the diagnosis of Alport syndrome with a high sensitivity and specificity (> 90%) [7, 23, 24]. High-throughput next-generation sequencing (NGS) technology can improve the diagnosis of Alport syndrome by providing molecular confirmation of *COL4A3*, *COL4A4*, or *COL4A5* mutations. We performed whole-exome sequencing for four individuals (II:1, II:2, III:1, and III:2); however, we did not identify any variant that could be possibly disease-causing in the three type IV collagen genes. It is possible that a small proportion of variants may have been missed owing to unfavourable insurance coverage or for other reasons. Alternatively, there could be a rare deep intronic variant affecting splicing and only detectable by RNA analysis. In addition, pathogenic genes in families with Alport syndrome may not be confined to a few regions. Known *COL4A3*, *COL4A4*, and *COL4A5* genes are scattered throughout many exons, making it difficult to develop predictive genetic tests.

Notably, a marked C3 staining with the corresponding electron-dense deposits along the mesangium was detected by immunofluorescence and electron microscope analysis of the kidney biopsy in the proband (III:1). Serum C3 was reduced in his mother (II:1), but it was normal in the proband and other affected family members. Excessive glomerular C3 fragment deposition without immunoglobulin, mesangial proliferation and crescent formation showed some morphological features of C3GN. In addition, the proband and his mother were affected more severely than other members. In the pedigree, familial C3GN was suspicious. The findings above prompted us to further investigate the complement system in the index family. A broad range of genetic contributors are definitely implicated in the pathogenesis of C3GN [25–27], so we screened a set of complement genes.

We identified a heterozygous missense variant c.508G > A (p.V170M; rs201073457) in the complement regulatory gene *CFHR5* in three members of this family (I:1, II:1 and III:1). *CFHR5* colocalizes with complement-containing glomerular immune deposits in a variety of glomerular pathologic states [28]. Based on genetic studies, copy number variations in *CFHR5* are implicated in C3GN [8, 29, 30], but significant enrichment of disease-associated rare variants is less [31]. The c.508G > A in the gene *CFHR5* generates a nonsynonymous alteration at amino acid position 170 (Val in wild-type and Met in mutant), which is strictly conserved among organisms. In silico programs (SIFT, SNAP, PolyPhen-2, and Mutation Taster) independently indicated the *CFHR5* c.508G > A to be potential functional variant. The exact mechanism of complement regulation is unknown, but is probably related to the C3b binding capacity. Then, we simulated 3D homology-modelled structures, which indicated mutant-type *CFHR5* increased conformational stability and induced an increase in the C3b-binding affinity. Meanwhile, this alteration has been previously reported in a patient with aHUS [32] and verified to exhibit significantly higher C3b binding capacity compared with wild-type *CFHR5* in in vitro functional assays [33]. Further replication studies are important and additional functional studies are required to reveal the exact biologic impacts in C3GN.

The above computational and functional assessments indicated that *CFHR5* c.508G > A may have contributed to the C3GN phenotype in the proband. Then, we applied the ACMG standards for variant classification [13], based on typical types of evidence (e.g., population data, computational and predictive data, functional data, segregation data and de novo data). The *CFHR5* c.508G > A variant is clearly PP3 [multiple lines of computational evidence support a deleterious effect on the gene or gene product (conservation, evolutionary, splicing impact, etc.)], likely PS3 (well-established in vitro or in vivo functional studies supportive of a damaging effect on the gene or gene product). Unfortunately, the evidence in favour of this variant being ‘pathogenic’ or ‘likely pathogenic’ is not substantial.

Meanwhile, it is not to be ignored that, C3GN is an ultra-rare kidney disease with an incidence of approximately one case per million per year [34]. Compared across ethnicities, the *CFHR5* c.508G > A variant is relatively frequent in East Asians (1000 Genomes: 0.6%), especially in the Chinese Han population (1000 Genomes: 1%). Though genetic investigation revealed the uncommon *CFHR5* missense variant in three affected individuals: the proband, his mother and his maternal grandma, the last two did not undergo renal biopsy. Currently, it is clearly BS1 (allele frequency is greater than expected for disorder), and clearly BS4 (lack of segregation in affected members of a family), which comes out that the *CFHR5* c.508G > A variant is classified as ‘benign’.

## Conclusions

Our study reports a pedigree characterized by microscopic haematuria and variety in the speed of onset of renal failure. The diagnosis of Alport syndrome is confirmed in the proband (III:1), his mother (II:1) and his maternal aunt’s daughter (III:2). Most notably, the proband revealed morphological features of C3GN based on genetic complement dysregulation background with concurrent histological evidence of Alport syndrome. Cases of Alport syndrome complicated with C3GN has never been reported, and underlying overlap requires further study.

## Supplementary Information

**Additional file 1: Table S1.** Primers of Sanger sequencing for CFHR5 c.508G > A.

## Data Availability

The datasets used and/or analysed during the current study are contained within the manuscript and the appendix, and available from the corresponding author on reasonable request. The totality of the data cannot be shared based on patient confidentiality concerns.

## Abbreviations

*CFHR5*: Complement factor H-related protein 5
C3GN: C3 glomerulonephritis
ESRD: End-stage renal disease
eGFR: Estimated glomerular filtration rate
MAF: Minor allele frequency
BUN: Blood urea nitrogen
sCr: Serum creatinine
GBM: Glomerular basement membrane
BM: Basement membrane
NGS: Next-generation sequencing
CKD: Chronic kidney disease
WT: Wild type
MT: Mutant type
