# Supplementary material for: Co-existence of Alport syndrome and C3 glomerulonephritis in a proband with family history
Source: Eur J Med Res. 2021 Jul 8;26:71. doi: 10.1186/s40001-021-00543-5 (PMC8265006; doi:10.1186/s40001-021-00543-5)
Supplement: Supplementary file 1 — Additional file 1: Table S1. Primers of Sanger sequencing for CFHR5 c.508G > A. [file 40001_2021_543_MOESM1_ESM.docx]

**Supplementary information for:**

**Co-existence of Alport syndrome and C3 glomerulonephritis in a proband with family history**

Yin Ding^1^, Xuanli Tang^1^, Yuanyuan Du^1^, Hongyu Chen^1^, Dongrong Yu^1^, Bin Zhu^1^ and Bohan Yuan^1^*

1 Department of Nephrology (Key laboratory of Management of Kidney Disease in Zhejiang Province), Hangzhou TCM Hospital Affiliated to Zhejiang Chinese Medical University, Tiyuchang Road 453, Hangzhou 310007, People’s Republic of China

*To whom correspondence and request for materials should be addressed: Bohan Yuan, Department of Nephrology (Key laboratory of Management of Kidney Disease in Zhejiang Province), Hangzhou TCM Hospital Affiliated to Zhejiang Chinese Medical University, Tiyuchang Road 453, Hangzhou 310007, People’s Republic of China, Email: [yuanbohan26@163.com](mailto:yuanbohan26@163.com).

**Supplementary Tables (on following pages)**

**• Supplementary Table S1** Primers of Sanger sequencing for CFHR5 c.508G>A

| **Supplemental Table S1** Primers of Sanger sequencing for CFHR5 c.508G>A | | | |
| --- | --- | --- | --- |
|  | **Forward primer** | **Reverse primer** | **PCR product size (bp)** |
| CFHR5 c.508G>A | TAGTCTATATGTTTTCTCGAAGGCA | ATTACTCCTTGATTACGGACACC | 503 |
